# Type II Heparin-Induced Thrombocytopenia: An Underrecognized Cause of Dialysis Catheter Dysfunction - A Case Report

**DOI:** 10.7759/cureus.41812

**Published:** 2023-07-13

**Authors:** Sailesh Karki, Binit Aryal, Arjun Mainali, Navodita Uprety, Kalpana Panigrahi, Samaj Adhikari

**Affiliations:** 1 Internal Medicine, Interfaith Medical Center, Brooklyn, USA; 2 Internal Medicine, One Brooklyn Health, Interfaith Medical Center, Brooklyn, USA

**Keywords:** subcutaneous unfractionated heparin, unfractionated heparin, hypercoagulable state, hypercoagulable, thromboembolic events, dialysis catheter thrombosis, dialysis catheter dysfunction, thrombocytopenia, heparin induced thrombocytopenia (hit)

## Abstract

Heparin-induced thrombocytopenia (HIT) is categorized into type 1 and type 2. It causes a decrease in platelet count during or shortly after exposure to heparin. Type 1 is mild and has a non-immune mechanism. Type 2 is a hypercoagulable state resulting from anti-heparin platelet factor 4 (PF4) IgG antibodies. These antibodies cause the activation of endothelium and thrombin generation. Type 2 HIT is complicated by life-threatening thromboembolic events such as deep venous thrombosis, pulmonary embolism, and myocardial infarction. HIT remains an under-recognized cause of dialysis catheter dysfunction and thrombosis. We present a case of a 66-year-old male with recurrent dialysis catheter thrombosis secondary to Type 2 HIT. Avoiding heparin-based dialysis or switching to non-heparin-based anticoagulation or peritoneal dialysis are the possible management strategies for such patients.

## Introduction

Heparin-induced thrombocytopenia [HIT] is categorized into type 1 and type 2 [1]. Type 1 HIT is characterized by a mild, transient non-immune drop in platelet count occurring in approximately 10% of patients [2]. In contrast, type 2 HIT is a prothrombotic state induced by platelet and endothelial activation, thrombin generation, and hypercoagulability [1,2]. Unfractionated heparin carries a higher risk of developing HIT than low molecular weight heparin, pertaining to its higher molecular weight and affinity to form the antigen-antibody complex [3,4]. HIT causes both arterial and venous thromboembolic complications such as deep venous thrombosis, pulmonary embolism, myocardial infarction, thrombotic stroke, occlusion of limb artery leading to amputation, upper-extremity venous thrombosis and skin necrosis [1-5]. However, HIT remains an under-recognized cause of dialysis catheter dysfunction and thrombosis. We present a case of a middle-aged male with recurrent thrombosis of intravascular femoral dialysis catheter secondary to type 2 HIT.

## Case presentation

A 66-year-old African-American male presented to the ED with a complaint of recurrent vomiting and watery, non-bloody diarrhea for three days. He denied abdominal pain, fever, dysuria, cough, or shortness of breath. He has a past medical history of HTN non-compliant with medication and subarachnoid hemorrhage with anterior communicating artery aneurysm. The patient endorsed that he had dental extraction three weeks back, for which he had been taking antibiotics and Ibuprofen for 10 days. He reported recreational use of cocaine. His family history was significant for end-stage kidney disease (ESRD) in his half-brother. On presentation to the emergency department, his vital signs were a blood pressure (BP) of 182/73 mm of Hg, heart rate of 67 per minute, temperature of 97.1 degrees Fahrenheit, respiratory rate of 20 per minute, and SPO2 of 98% on room air. Physical examination was unremarkable except for bilateral lower extremity pitting edema. The abdomen was soft and non-tender. Laboratory findings were notable for blood urea nitrogen (BUN) of 77 mg/dL and creatinine of 10.4 mg/dL. BUN: Cr one year before admission was 22/1.4 mg/dL. Phosphorus was 4.4 mg/dL, and calcium was 6.8 mg/dL, with an albumin level of 1.7 g/dL. A complete blood count (CBC) revealed normocytic anemia with a hemoglobin of 10.9 g/dL, hematocrit of 32.1%, and mean corpuscular volume (MCV) of 86.5 fL. The platelet count on admission was 232,000 per microlite.

**Figure 1:**
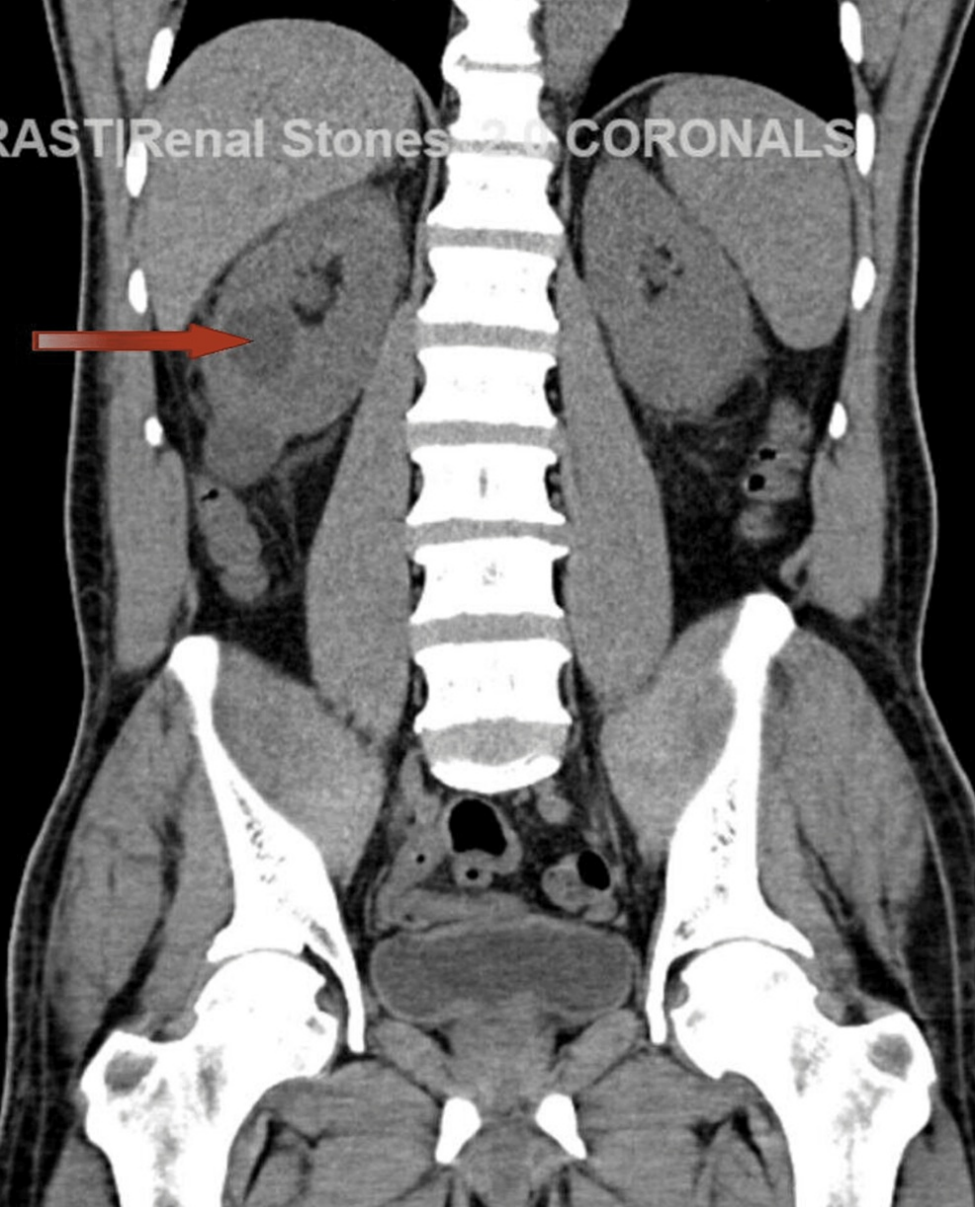
Computed tomography (CT) of the abdomen and pelvis in coronal section shows renal cyst on the right side (red arrow)

Pertinent laboratory parameters at presentation are depicted in Table 1. CT abdomen and pelvis revealed several cysts in the right kidney with the largest in the mid-pole measuring 3.4 x 3.0 cm as shown in Figure 1. No splenomegaly was noted.

**Table 1:** Laboratory parameters at the presentation WBC - white blood cell count, BUN - blood urea nitrogen, AST - aspartate transaminase, ALT - alanine transaminase, ALP - alkaline phosphatase, INR - international normalized ratio

Test	Ref range and units	Values
WBC	4.5-11.0 10x3/uL	4.9
Neutrophil %	40.0-70.0 %	54.5
Lymphocytes %	22.0-48.0 %	35.9
Monocytes %	2.0-14.0 %	8.0
Eosinophil %	0.5-5.0 %	1.2
Basophil %	0.0-2.0 %	0.2
Hemoglobin	11.0-15.0 g/dL	10.7
BUN	7.0-18.7 mg/dL	82.2
Creatinine	0.57-1.11 mg/dL	10.59
Sodium	136-145 mmol/L	135
Potassium	3.5-5.1 mmol/L	4.4
CO2	22-29 mmol/L	20
Total bilirubin	0.2-1.2 mg/dL	0.7
ALT	10-55 U/L	13
AST	5-34 U/L	20
ALP	40-150 U/L	125.5
Albumin	3.5-5.2 g/dL	1.7

**Figure 2:**
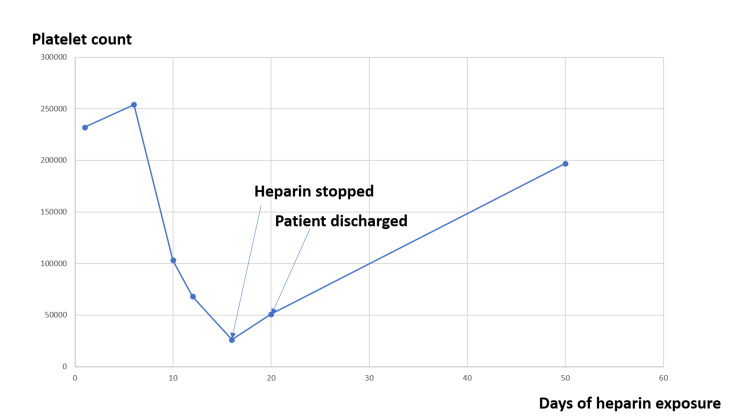
Platelet count vs. day of heparin exposure

A temporary femoral temporary dialysis catheter was placed and hemodialysis was initiated. The patient was started on unfractionated heparin 5000 units subcutaneously twice daily for deep vein thrombosis (DVT) prophylaxis. Platelet count started dropping on day 10 of admission (103,000) and on day 12 of admission (68,000) and progressively decreased to 26,000 on day 16 of admission. The trend of platelets during the hospital stay is demonstrated in Figure 2. 

Workup for thrombocytopenia showed serum vitamin B12 of 374 pg/mL (ref range 232-1245 pg/mL) and folate of 8.2 ng/mL (ref range >3 ng/mL). Blood cultures were negative for any growth. The patient had multiple episodes of catheter thrombosis starting from day six of admission. Multiple attempts were made to resolve the malfunction with alteplase injection and manipulation of the catheter, in which extensive clots were noted in the catheter. However, the patient had a persistent malfunction of the dialysis catheter. Fluoroscopy showed attenuated flow with thrombosis in the catheter. Vascular dopplers for deep vein thrombosis were negative on bilateral upper and lower extremities. Venocavagram revealed no thrombus in the right common iliac vein or inferior vena cava. The 4T score for the patient was seven (two points for thrombocytopenia, one point for timing of platelet fall, two points for thrombosis, and one point for other apparent causes), prompting immediate HIT workup. Heparin-induced platelet antibody was found to be 3.296 OD (ref range 0.0-0.4 OD), strongly suggestive of HIT. He was started on apixaban 2.5 mg twice a day for anticoagulation after the positive result, as per the opinion of the hematologist. Serotonin release assay was positive, confirming HIT (73% with low dose heparin (Ref range 0-20%) and <1 with high dose heparin(Reference range 0-20%)). The patient underwent permacath placement for dialysis. Administration of heparin was stopped, and his platelet counts improved gradually. In subsequent dialysis through permcath, heparin was avoided. The patient did not have any further episodes of thrombosis during the hospitalization, and he was discharged with a platelet count of 51,000 per microliter. On a follow-up visit, one month after discharge, the platelet count was found to have recovered to 197,000 per microliter.

## Discussion

Our patient has a unique occurrence of an isolated dialysis catheter thrombosis secondary to HIT without any evidence of deep vein thrombosis of the extremities. Considering the routine use of heparin in hemodialysis filters, early and accurate diagnosis of HIT in patients with vascular access site thrombosis is crucial in preventing severe morbidity in hemodialysis patients. Along with the general complications of HIT, various complications specific to dialysis patients have been reported, such as clotted upper extremity arteriovenous grafts and bilateral subclavian vein stenosis as reported by Pham et al. [6] and dialysis circuit clotting, as well as catheter clotting, reported in a case series by Gameiro et al. [7].

Antibodies against platelet factor 4 in patients without HIT have been investigated in various studies [8-11]. Heparin-induced antibodies without any associated thrombocytopenia have been found in higher prevalence in patients undergoing hemodialysis compared to controls [8]. An association between repeated early hemofiltration-filter clotting was shown with the presence of anti-PF4/heparin antibodies, irrespective of the platelet count in a retrospective study [9]. A study from Shea et al. showed no correlation between vascular access site thrombosis and anti-PF4 antibody titer in patients without HIT [10]. However, a prospective study by Vega et al. demonstrated increased cardiovascular and all-cause mortality in patients with a higher titer of HIT antibody [11]. These studies support the necessity for further studies investigating anti-PF4 antibody titers as a prognostic marker and the need to interpret anti-PF4 titers in the appropriate clinical context. 

In our patient, the platelet count remained stable after unfractionated heparin was stopped, the catheter was removed, and the patient received apixaban. In subsequent dialysis through permcath, heparin was avoided. As in our patient, Sidra et al. reported stable platelet counts with the use of apixaban [12]. Theodore et al.'s prospective study on patients with acute HIT treated with rivaroxaban as a primary therapy found that the patients recovered without new, progressive, or recurrent thrombosis [13]. The degree of simplicity offered by oral direct oral anticoagulants (DOACs) in the treatment of acute HIT is of high importance, and DOACs have been recommended as an option for the treatment of HIT by the American Society of Hematology (ASH) HIT guidelines [14].

## Conclusions

Thrombocytopenia is common and multifactorial in hospitalized patients. However, making the early diagnosis of HIT demands a high index of suspicion. Our case highlights the importance of prompt investigation directed to rule out HIT in a patient with recurrent dialysis catheter thrombosis and thrombocytopenia before the development of arterial or venous thrombosis. Early recognition followed by investigation for HIT antibodies, prompt cessation of heparin, and use of non-heparin anticoagulation remains the cornerstone to prevent morbidity and mortality of HIT.
